# Defining levels of dengue virus serotype-specific neutralizing antibodies induced by a live attenuated tetravalent dengue vaccine (TAK-003)

**DOI:** 10.1371/journal.pntd.0009258

**Published:** 2021-03-12

**Authors:** Laura J. White, Ellen F. Young, Mark J. Stoops, Sandra R. Henein, Elizabeth C. Adams, Ralph S. Baric, Aravinda M. de Silva

**Affiliations:** 1 Department of Microbiology and Immunology, The University of North Carolina at Chapel Hill School of Medicine, Chapel Hill, NC, United States of America; 2 Department of Epidemiology, The University of North Carolina at Chapel Hill School of Public Health, Chapel Hill, NC, United States of America; La Jolla Institute for Allergy and Immunology, UNITED STATES

## Abstract

The four dengue virus serotypes (DENV1-4) infect several hundred million people each year living in tropical and sub-tropical regions. Clinical development of DENV vaccines is difficult because immunity to a single serotype increases risk of severe disease during a second infection with a new serotype. Leading vaccines are based on tetravalent formulations to induce simultaneous and balanced protective immunity to all 4 serotypes. TAK-003 is a tetravalent live attenuated dengue vaccine candidate developed by Takeda Vaccines Inc, which is currently being evaluated in phase 3 efficacy trials. Here, we use antibody depletion methods and chimeric, epitope transplant DENVs to characterize the specificity of neutralizing antibodies in dengue-naïve adults and non-human primates immunized with TAK-003. Our results demonstrate that TAK-003 induced high levels of DENV2 neutralizing antibodies that recognized unique (type-specific) epitopes on DENV2. In contrast, most vaccinated subjects developed lower levels of DENV1, DENV3 and DENV4 neutralizing antibodies that mainly targeted epitopes that were conserved (cross-reactive) between serotypes.

**Trial Registration:** ClinicalTrials.gov NCT02425098.

## Author summary

The licensed tetravalent dengue vaccine Dengvaxia is indicated for individuals with previous exposure to dengue. In subjects with no past dengue infection, vaccine trials showed low efficacy against some serotypes and increased risk of severe disease upon post-vaccination infection. The development of tetravalent dengue vaccines has been guided by neutralizing antibodies to each serotype as a measure of safe and effective immunity. However, the absolute level of neutralizing antibodies to each serotype has proven to be an unreliable correlate of protection. Recent studies suggest that a better correlate may be levels of antibodies to epitopes that are unique to each serotype and are independently stimulated by each vaccine component, rather than total quantity of neutralizing antibodies. Here, we mapped the antibody specificity induced by the Takeda tetravalent dengue vaccine TAK-003 in monkeys and humans with no prior immunity to dengue. The vaccine induces high levels of dengue serotype 2 specific neutralizing antibodies that map to known protective epitopes. In contrast, the dengue serotype 1, 3 and 4 specific responses are lower and predominantly consist of cross-reactive antibodies binding to antigenic regions conserved between serotypes. It remains to be determined whether these cross-reactive antibodies, most likely induced by the serotype 2 component, contribute to long-term protection after vaccination.

## Introduction

The four dengue virus serotypes (DENV1-4) infect several hundred million people each year living in tropical and sub-tropical regions [1–3]. Clinically, DENV infections can be inapparent or present as a febrile illness that may or may not progress to severe dengue hemorrhagic fever and shock syndrome. Primary infection with one DENV serotype leads to long-term protective immunity against the homologous serotype and transient cross protection against other serotypes [4,5]. DENV serotype-specific neutralizing antibodies, which circulate for life, are correlated with durable protection against re-infection by the same serotype [6–8]. Individuals experiencing second DENV infections with heterologous serotypes are at greater risk for developing severe dengue hemorrhagic fever and shock syndrome compared to individuals experiencing their first infection. The ability of some DENV serotype cross-reactive antibodies to promote the entry of heterologous serotypes into Fc receptor-bearing target cells is widely supported as the initiating event that culminates in severe disease [9,10]. To minimize the risk of DENV vaccines inducing antibodies that enhance DENV infections, leading vaccines are based on tetravalent formulations to induce simultaneous and balanced protective immunity to all 4 serotypes. However, in practice, it has been challenging to achieve balanced replication and immunity with tetravalent DENV vaccines. Here we characterize the properties of neutralizing antibodies (nAbs) induced by a leading live attenuated tetravalent DENV vaccine (TAK-003) to determine the contribution of each vaccine component to the serotype-specific neutralizing antibody response.

After a primary DENV infection the durable protective immune response is directed to serotype-specific epitopes on the infecting virus, leaving individuals fully susceptible to a second infection with a new serotype. After a second infection with a new serotype, the protective immune response is mainly directed to epitopes conserved between serotypes, and clinically significant tertiary DENV infections are rare [11,12]. While the immune mechanisms responsible for serotype cross-protective immunity after a second infection have not been full elucidated, immunological memory from the first infection is critical for establishing durable cross-protective immunity. Recent studies indicate that reactivation of memory cells by a second infection selects for high affinity, cross-reactive antibodies and T cells responsible for cross-protective immunity [13–15]. These studies are relevant to understanding how live attenuated tetravalent DENV vaccines will protect people [16]. In those who have experienced a DENV infection before receiving the vaccine, even a poorly balanced vaccine dominated by one vaccine serotype is likely to induce cross protective immunity by activating memory. In DENV naïve individuals with no immune memory, the protective immune response to each serotype will strongly depend on how well each vaccine component independently performs.

There are several DENV vaccines under development, including two live attenuated tetravalent DENV vaccines (TAK-003 developed by Takeda Vaccines Inc. and TV-003 developed by the US National Institutes of Health) currently in phase III clinical trials and one live attenuated tetravalent vaccine, Dengvaxia, developed by Sanofi Pasteur that has been licensed for use in children with pre-existing immunity to DENV [17–19]. In children with no immunity to DENVs, Dengvaxia was poorly efficacious and the vaccine increased the risk of severe dengue disease upon exposure to wild type DENV infections. The poor performance of Dengvaxia in dengue-naïve children is most likely due to unbalanced replication of vaccine components, where the DENV4 component is replication- and immune-dominant compared to the other three serotypes [20–24], although reduced dengue specific T cell antigens may also play a role.

TAK-003 developed by Takeda Vaccines Inc. is based on an attenuated DENV2 strain (TDV-2) and 3 additional chimeras of the attenuated DENV2 strain expressing the envelope proteins of DENV1, 3 and 4 (TDV-1, TDV-3 and TDV-4). This candidate vaccine, which has been shown to be safe and immunogenic in phase 1 and 2 trials, is currently being evaluated in a large multi-country phase 3 efficacy trial [25,26]. In preclinical and early clinical studies, the DENV2 component of the vaccine was observed to replicate better than the other three chimeric components [27–29]. In people with no pre-existing immunity to DENVs, TAK-003 stimulated higher levels of neutralizing antibody (nAb) to DENV2 compared to the other three serotypes [25,26]. While TAK-003 stimulates lower levels of neutralizing antibodies to DENV1, 3 and 4, it is unclear if these antibodies target epitopes that are conserved across DENV serotypes (cross-reactive) or epitopes that are unique (type-specific) to each serotype. Neutralizing antibodies directed to type-specific epitopes are indicative of a vaccine component replicating and independently stimulating antibodies to a DENV serotype. Here, we describe the levels of DENV serotype-specific and cross-reactive neutralizing antibodies in dengue-naïve adults and non-human primates immunized with TAK-003.

## Materials and methods

### Ethics statement

This study used deidentified human samples under UNC IRB exemption protocol. Serum samples were collected by Takeda Vaccines Inc. in the completed Phase 2 clinical trial DEN-205 (ClinicalTrials.gov Identifier: NCT02425098).

### Cell lines and viruses

C6/36 *Aedes albopictus* mosquito cells were used to grow DENV1-4 to use in ELISA and virus neutralization assays. Cells were maintained in minimal essential medium (MEM; Gibco) at 32°C. Vero-81 mammalian cells (American Type Culture Collection; CCL-81) were used to grow DENV1-4 for the generation of purified antigens. Vero cells were maintained in Dulbecco’s modified Eagle’s medium-F12 (DMEM-F12) at 37°C. All growth and maintenance media used were supplemented with 5% fetal bovine serum (FBS), 100 U/mL penicillin, 100 mg/mL streptomycin, 0.1 mM non-essential amino acids (Gibco), and 2 mM glutamine. All cells were incubated in the presence of 5% CO2. The 5% FBS was reduced to 2% to make infection medium for each cell line. DENV1 (American genotype; strain West Pac74), DENV2 (Asian genotype; strain S-16803), DENV3 (Asian genotype; strain CH-53489), and DENV4 (American genotype; strain TVP-376) (provided by Robert Putnak, Walter Reed Army Institute of Research, Silver Spring, MD) were used for both binding enzyme-linked immunosorbent assays (ELISAs), neutralization assays, and depletion assays, unless otherwise noted. DENV1-4 coupled to Dynabeads in depletion assays were grown in Vero-81 cells and purified by tangential flow concentration and density gradient ultracentrifugation. All the recombinant chimeric viruses and WT infectious clones used here were constructed at UNC using a cuadripartite complementary DNA clone, as reported earlier [30].

### Vaccine formulations

The DEN-205 subjects included in this study received one of two TAK-003 vaccine formulations. An earlier formulation refered as HD-TDV contained 2 X 10^4^ plaque-forming units (pfu) of TDV-1, 5 X 10^4^ pfu of TDV-2, 1 X 10^5^ pfu of TDV-3, and 3 X 10^5^ pfu of TDV-4. A new formulation, refered as TDV, contained identical dosages of TDV-1, TDV-3, and TDV-4, but a lower dosage of TDV- 2 (5 X 10^3^ pfu) (S1 Table) [29].

### Source of non-human primates serum samples

The NHP sera used in this study came from Takeda study DNHP-007. The study was designed to determine immunogenicity induced after one or two doses of the tetravalent TAK-003 vaccine (3.17x10^4^ pfu TDV-1, 9.72 x10^4^pfu TDV-2, 3.75 x10^5^ pfu TDV-3 and 3.65 x10^5^ pfu TDV-4). Here we studied samples from 30 animals collected 180 days after one or 2 doses of TAK-003. We also studied the antibodies in the sera of 18 control unvaccinated animals, collected 180 days after subcutaneous challenge with WT DENV1 strain West Pac74, DENV2 strain New Guinea C or DENV3 strain Sleman 78, for comparison of serotype-specific antibodies induced after primary infections and after tetravalent vaccination. The number of animals in each group is listed in S2 Table.

### Source of human serum samples

Subjects in the Phase 2 clinical trial DEN-205 were randomly assigned 1:1 to receive a single dose of either TDV or HD- TDV on Day 1 of the trial. Random assignment was stratified based on dengue IgG status (positive or negative) at screening, with a maximum of 200 subjects to be enrolled per dengue IgG status group [29]. We received from Takeda 30 baseline seronegative serum samples, including 14 TDV and 16 HD-TDV. Here we analyzed sera (N = 30) from seronegative subjects collected 180 days after vaccination (S3 Table). In addition, we analyzed baseline sera from 14 DENV participants in clinical trial DEN-205, collected on day 0 before vaccination. Based on DENV1-4 Neut50 titers, 6 subjects had a past primary DENV1 (DENV1 Neut50 titers > 4-fold higher than Neut50 titers to other 3 serotypes) and 8 subjects had a past primary DENV2 infection. The time between primary infection and sample collection was unknown, but the pattern of the neutralizing antibody titers to DENV1-4 suggest that the samples were from the convalescent phase (S4 Table).

The study reported here is not part of the primary or secondary objectives of the trial.

### Antibody depletions

We have developed antibody depletion methods to separate antibodies in polyclonal sera into cross-reactive (CR), which are directed to epitopes on the virion that are conserved among dengue virus serotypes, and serotype-specific Abs (TS), which bind unique epitopes on each serotype [12,24,31]. In principle, Abs with specific binding properties are removed from sera by incubation with purified whole virion antigen of the desired serotype covalently linked to polystyrene or magnetic beads. The properties of Abs in undepleted and Ab-depleted serum are then assessed by ELISA and micro-Focus Reduction Neutralization Test (mFRNT) assays.

Samples from NHP were depleted using purified DENV or BSA control protein adsorbed onto Polybeads polystyrene microspheres (Polysciences, Inc.) as reported earlier [12,24]. Samples from vaccinated and unvaccinated human subjects were depleted following a modified depletion protocol using magnetic beads and magnetc separation. Briefly, Magnetic Dynabeads M280 Tosyl activated (DB) (Invitrogen 14203, 14204) were first covalently linked to the dengue cross-reactive monoclonal antibody 1M7 following manufacturer’s protocol (50 ug 1M7 per 5 mg DB). DB-1M7 complex was blocked with BSA in PBS and then washed with 0.1 M 2-(*N*- morpholino) ethane sulfonic acid (MES) buffer, and then incubated for 1 h at 37°C in separate with purified DENV1, DENV2, DENV3, DENV4 or Bovine Serum Albumin (BSA) antigen control at a ratio of 100 ug of antigen per 5 mg DB. After 3 PBS washes the bound antigen was cross-linked to the DB-1M7 complex with 2% PFA for 20 min at room temperature. Serum samples were diluted 1:10 and incubated with the Antigen-DB complex for 1 h at 37°C with end-over-end mixing under 3 conditions: a) BSA-DB as undepleted control (total nAbs, TS+CR) (UND), b) Heterologous serotypes-DB to remove CR Abs and leaves TS nAbs, (HET) and c) Homologous serotype-DB, as indicator of depletion efficiency (HOM). Abs bound to beads were magnetically separated from the depleted serum. For the analysis of clinical samples, a paired depletion approach was used, where after DV1-DB+DV3-BD heterologous depletions (HET) of serum, the remaining titers to DV2 and DV4 represented the fraction of TS nAbs to DV2 and DV4 respectively. On the other hand, remaining Neut50 titers to DV1 and DV3 after depletions with DV2-DB+DV4-DB (HET) represent the fraction of TS nAbs to DV1 and DV3. Homotypic depletion (HOM) efficiency was determined for each serotype by ELISA and mFRNT. For the analysis of preclinical samples, HET depletion was performed with DENV2 for measuring TS nAbs to DENV1 and DENV3. To measure TS nAbs to DENV2, HET depletion was performed with a mix of DB conjugated to DENV1, DENV3 and DENV4. Three rounds of depletion were usually needed for successful removal of >80% of homologous dengue Abs.

### Analysis of TS nAbs

Neut50 titers in the undepleted, homotypic-depleted and heterotypic-depleted sera were determined by mFRNT. The fraction of Abs in the sera that were TS was calculated using two independent methods. Method 1 used the formula: %TS nAbs = [Neut50 HET depletion–Neut50 HOM depletion]/[Neut50 BSA depletion–Neut50 HOM depletion] x 100, as reported earlier [24]. Method 2 used more stringent inclusion criteria for TS nAbs analysis, and only samples with heterologous depletion efficiency > 90% or HET depleted titers <20 were included. Samples with BSA-depleted titers <40 were considered negative for TS nAbs. Log10 transformed values of each Neut50 titer were used to calculate levels of TS nAbs using the equation = [(log10 Neut50 HET depletion/log10 Neut50 BSA depletion) x100]. A TS NAb value above 55 was selected as cutoff for evidence of TS nAbs.

### ELISA

Antigen capture ELISA was used to confirm efficient depletion of targeted antibodies. Briefly, purified antigen was plated at 100ng/well in a 96 well ELISA plates overnight at 4°C. Plates were blocked with 3% (vol/vol) normal goat serum (Gibco, Thermo Fisher, USA)-Tris buffered saline (TBS) -0.05% (vol/vol) Tween 20 (blocking buffer). Depleted sera were diluted and added to the antigen coated ELISA plates. Alkaline phosphatase-conjugated secondary Abs were used to detect binding of sera with p- nitrophenyl phosphate substrate, and reaction color changes were quantified by spectrophotometry at 405nm.

### Titration of wild-type (WT) and chimeric viruses on Vero cells

For titrations of WT and chimeric viruses, viral stocks were diluted 10-fold serially in dilution medium (OptiMEM, Grand Island, NY) supplemented with 2% heat-inactivated fetal bovine serum (HI-FBS; Hyclone Defined) and 1x antibiotic/antimycotic. Following a 1hr infection, cells and inoculum were overlaid with overlay medium: OptiMem (Gibco, Grand Island, NY) containing 5% carboxymethylcellulose, 2% HI-FBS (Hyclone defined), and 1x antibiotic/antibiotic (Gibco, Grand Island, NY). Following a 2 day incubation (to achieve countable foci of infection), cell monolayers were washed with 1xPBS followed by fixation/permeabilization with 4% paraformaldehyde/ 0.01% saponin (v:v). Fixed cell monolayers were blocked in a solution of PBS containing 5% non-fat milk and incubated with a primary antibody cocktail of 4G2 and 2H2 murine mAbs. Following thorough PBS washes, infectious foci were visualized using HRP-goat-anti-mouse secondary antibody (KPL, Gaithersburg, MD), followed by TrueBlue substrate and foci were enumerated and used to calculate infectious titer. Number and size of foci were analyzed with a CTL Immunospot instrument.

### DENV neutralization assay

To measure neutralizing antibody titers in un-depleted and depleted sera, we used a micro focus reduction neutralization test adapted to a 96-well plate format (mFRNT) as described earlier [32]. Briefly, 96-well plates were plated with 2 x 10^4^ Vero-81 cells/well and incubated at 37°C for 24 hours. Serial 3-fold dilutions of each serum were mixed with 50–100 FFU of virus in DMEM with 2% FBS. The virus-Ab mixtures were incubated for 1 h at 37°C and then transferred to the confluent monolayer of Vero 81 cells on the 96-well plates. Following an additional 1 hour incubation at 37°C, the monolayers were overlaid with Opti-MEM (Gibco, Grand Island, NY) containing 2% FBS and 1% (wt/vol) carboxymethyl cellulose (Sigma, St. Louis, MO). EC50 values were calculated by graphing % neutralization vs. serum dilution and fitting a sigmoidal dose response (variable slope) using Prism 8 (GraphPad Software, San Diego, CA, USA). Neut50 titers represent the dilution at which the serum neutralizes 50% of the infection. Log transformed data from Neu50 values were used to calculate GMT +/- 95%CI. Criteria to accept values to be reported were an R^2^ >0.75 and a Hill Slope >0.5 absolute value.

### Statistical analysis

Variation between groups was measured by a non-parametric Friedman test with Dunn’s multiple comparisons, and by Mann-Whitney test. P < 0.05 were considered statistically significant.

## Results

### Levels of DENV serotype specific nAbs in nonhuman primates after TAK-003 vaccination

Non-human primates (NHP) have been useful models to evaluate the immunogenicity and protective efficacy of dengue vaccines [27,33]. Here we used NHPs sera from Takeda study DNHP-007 (S2 Table) to characterize antibodies induced by TAK-003 vaccination and WT DENV infection. The monkeys were vaccinated with one (N = 4) or two doses (N = 4) of the TAK = 003 or infected with WT DENV1, 2 or 3 (N = 6 per serotype). Serum was collected 180 days after the last vaccine dose or WT DENV infection. The immune sera were pre-incubated with magnetic beads coated with different DENV serotypes to remove DENV serotype cross reactive antibodies, while retaining any type-specific antibodies to the serotype of interest.

**Fig 1:**
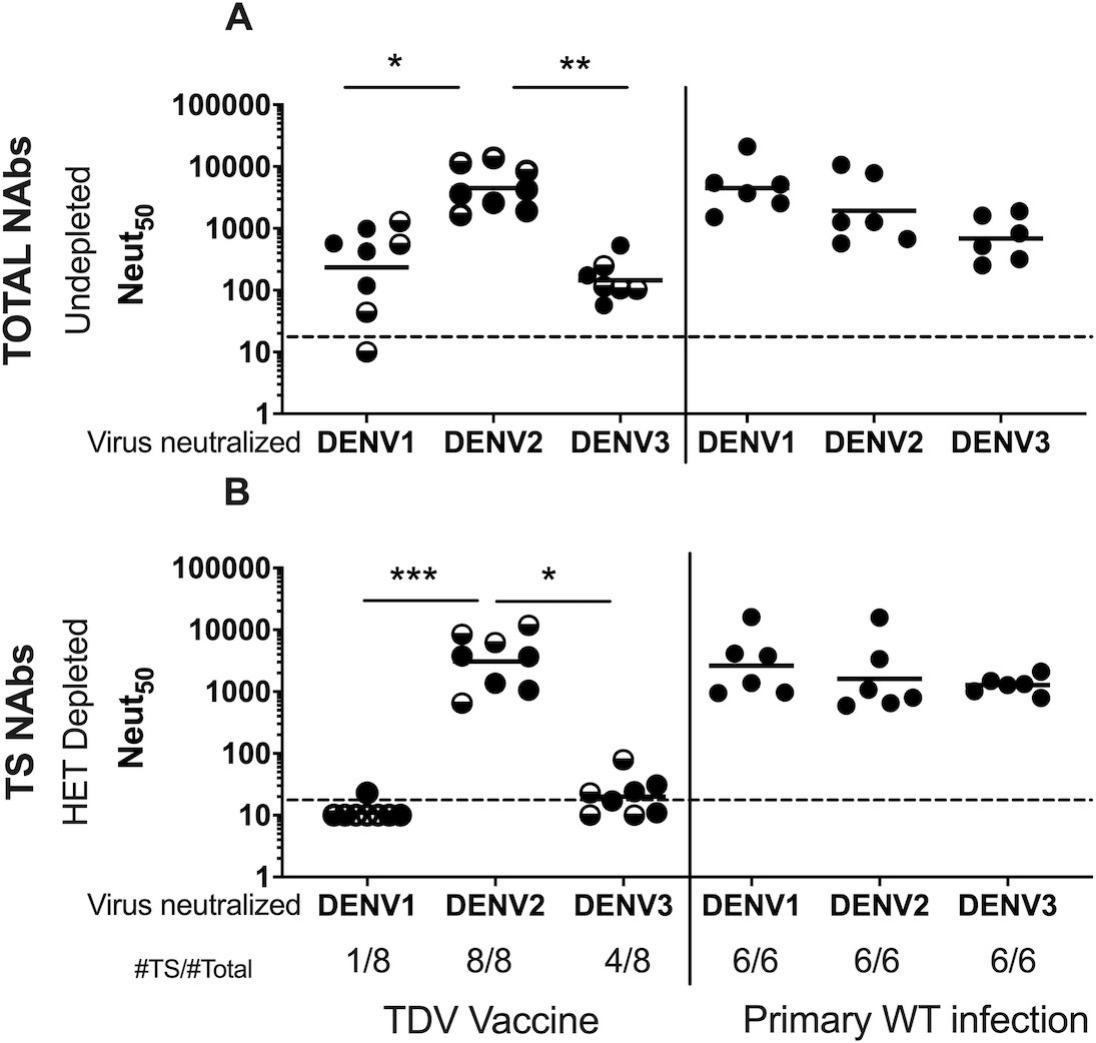
**50% Neutralization titers (Neut**_**50**_**) in undepleted (A) and Heterologous virus-depleted (B) baseline seronegative NHP vaccinated with TDV (left) or infected with WT DV1, DV2 or DV3 (right).** Non-human primates were vaccinated with one dose (filled circles) or two doses (half filled circles) of TDV, or infected once with wild type DV1, DV2 or DV3 (S2 Table). Animals were bled 180 days after infection or last vaccination. Each sample underwent BSA-depletion (undepleted control) (A), depletion of cross-reactive nAbs by incubation with heterologous serotype viruses (HET depleted) (B) and depletion with the homologous serotype, before determining Neut50 titers by mFRNT assay. Each value represents the titer after subtraction of the background homologous-depletion titer. Each dot represents one animal. Line represents the GMT of the Neut_50_ titers. The lowest serum dilution tested was 20 (horizontal dotted line). Statistical analysis was done using nonparametric Friedman test with Dunn’s multiple comparison among serotypes after TDV vaccine. * P < 0.05; ** P < 0,005; *** P < 0.0005.

As depicted in Fig 1, undepleted sera reflect total levels of nAbs to each serotype (Fig 1A), and HET depleted sera reflect levels of serotype specific nAbs (Fig 1B, HET Depleted). The vaccine induced total nAbs titers >20 to DENV1 in 7/8 animals (GMT = 233), to DENV2 in 8/8 (GMT = 4,489), and to DENV3 in 8/8 animals (GMT = 145). The titers to DENV2 were significantly higher than those to DENV1 and DENV3. After a second dose of TDV (half-filled symbols), a modest (<4-fold) increases in NAb titers to each serotype was seen in some animals, but there was no significant difference between one and two doses. Six months after infection with WT DENV1 (N = 6), DENV2 (N = 6) or DENV3 (N = 6), geometric mean titers were 4,503, 1,923, and 686 respectively.

Fig 1B shows the corresponding Neut_50_ titers after heterologous depletion, representing the fraction of induced nAbs binding to type-specific epitopes on each serotype. All animals showed evidence of DENV2 TS nAbs after HET depletion (GMT = 3,077), with the TS fraction for each animal ranging between 39 and 100% of total nAbs. For DENV1, only 1/8 animals had TS nAbs after HET depletion (Neut50 = 23). For DENV3, 4/8 had low levels of TS nAbs (GMT = 34).

In animals infected with WT DENV, a large proportion of TS nAbs remained after HET depletion with Neut50 GMTs of 2,614 for DENV1, 1611 for DENV2 and 1273 for DENV3 (Fig 1A and 1B, right panels).

We conclude that NHPs infected with WT DENV1, 2 or 3 develop high levels of nAbs that mainly target type-specific epitopes on each serotype. TAK-003 induced high levels of DENV2 TS nAbs, and this response was similar to that observed in NHPs infected with WT DENV2. TAK-003 induced low levels DENV1 and 3 nAbs that were mainly directed to serotype cross-reactive epitopes, and this response was fundamentally different from the response observed in monkeys infected with WT DENV1 or 3. In this NHP study, the response to DENV4 was not evaluated.

### Levels of DENV serotype specific nAbs in dengue-naïve humans after TAK-003 vaccination

**Fig 2:**
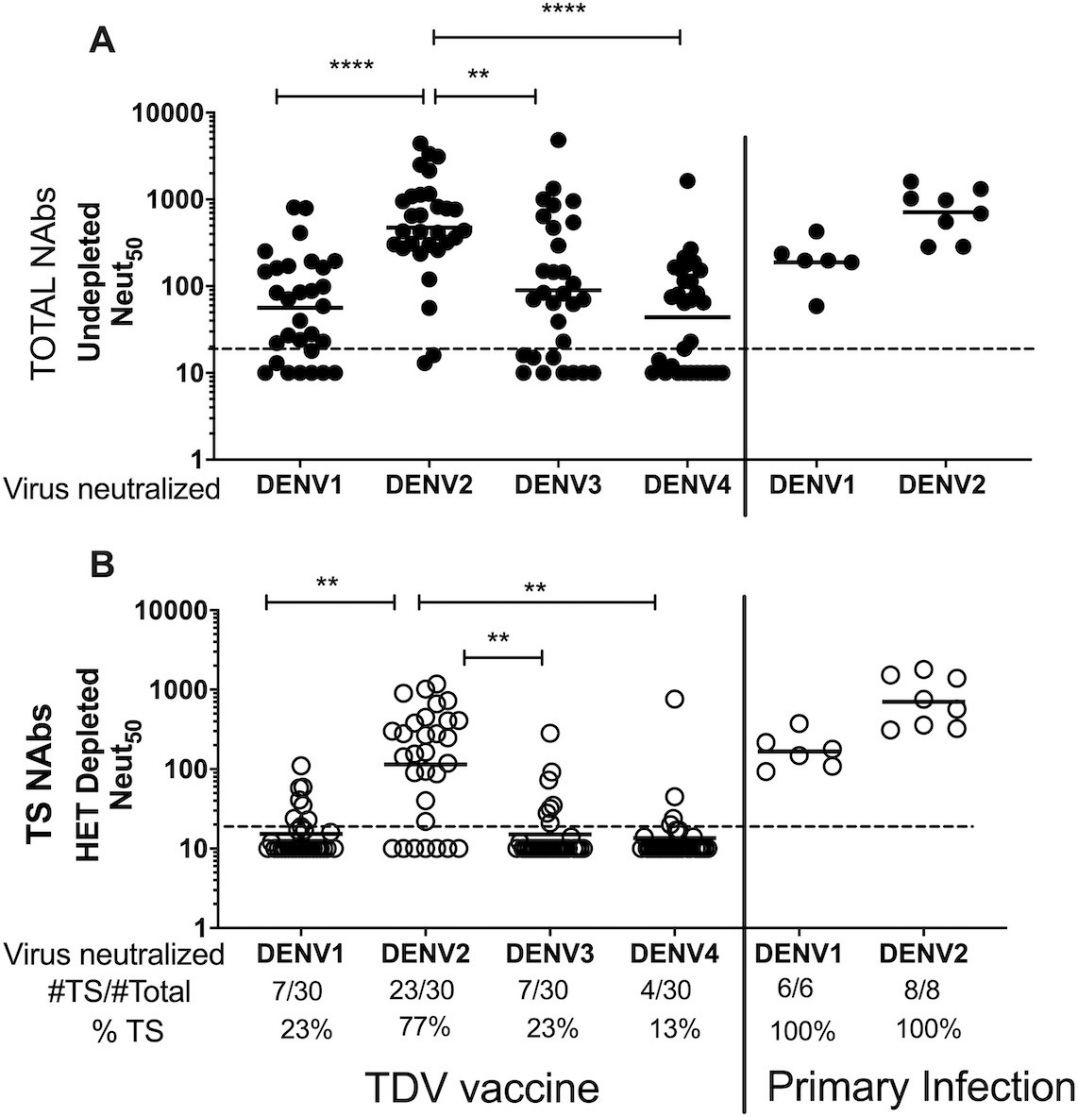
**50% Neutralization titers (Neut50) in undepleted (A) and Heterologous virus-depleted (B) baseline seronegative subjects vaccinated with one dose of TDV (left) or after exposure to primary DV1 or DV2 infection (right).** The TDV vaccine cohort included sera from 30 subjects collected 180 days after TDV vaccination. The primary infection cohort included 6 primary DENV1 and 8 primary DENV2, collected at baseline in study DEN-205. Each sample was BSA-depleted (Undepleted control) (A) or depleted of cross-reactive nAbs using heterologous serotype viruses (HET Depleted) (B). To measure DV1 and DV3 TS neutralization, HET depletion was done with a mix of DV2+DV4 dynabeads. For DV2 and DV4 TS titers, Het depletion was done with a mix of DV1+DV3 dynabeads. Each value represents the titer after subtraction of background homologous depletion titer. Each dot represents one subjects. Line represents the GMT of the Neut50 titers. The # of subjects with evidence of TS nAbs >20 after HET depletion/#total subjects analyzed is shown at the bottom of panel B. % TS is the percent of subjects with TS nAbs = or > 20. The lowest serum dilution tested was 20 (horizontal dotted line). Statistical analysis was done using nonparametric Friedman test for multiple comparison among serotypes after TDV vaccine. ** P < 0,005; **** P < 0.0001.

We obtained 30 vaccine immune sera from DENV naïve individuals enrolled in Takeda’s DEN-205 study, who received the vaccine (S3 Table). The samples were collected 180 days after vaccinating adults with one dose of TAK-003. nAbs to DENV1, 2, 3 and 4 were detected in 73% (GMT = 57), 93% (GMT = 474), 70% (GMT = 90) and 57% (GMT = 44) of subjects respectively (Fig 2A). The overall nAbs titers to DENV2 were significantly higher than those to the other 3 serotypes. Subjects had received one of two formulations, TDV (N = 14) or HD-TDV (N = 16), that differed in the dose of the TDV2 component, which was 10 times higher in TDV-HD. Unless otherwise indicated, data from both formulations were grouped together for analysis. When the two formulations, TDV and HD-TDV, were compared, we found that HD-TDV induced significantly higher Neut50 titers against DENV2, while no differences were found for DENV1, DENV3 and DENV4 (S5 Table). Fig 2B shows the nAb titers after HET depletion, providing a measure of TS nAbs stimulated by each vaccine component. Out of 22 DENV1 responders (subjects with DENV1 Neut50 titers >20), 15 subjects failed to neutralize DENV1 after cross-reactive Abs were depleted. Out of 28 subjects with DENV2 Neut50 titers >20, the majority (23) showed neutralization after depletion of CR Abs. DENV3 and DENV4 neutralization showed a pattern similar to DENV1, where most subjects failed to neutralize the corresponding serotype after removal of cross-reactive antibodies (Fig 2B). As controls, we analyzed immune sera from individuals exposed to past primary DENV1 (6 subjects) or DENV2 (8 subjects) infections before enrolling in study 205. All 14 subjects had high levels of total and TS nAbs to DENV1 or 2 (Fig 2A and 2B, right panels). These data is also presented as the proportion (%) of TS nAbs over Total nAbs for each serotype in S1 Fig. The raw Neut50 titers after depletions and the % depletion efficiency are shown in S6 Table.

**Fig 3:**
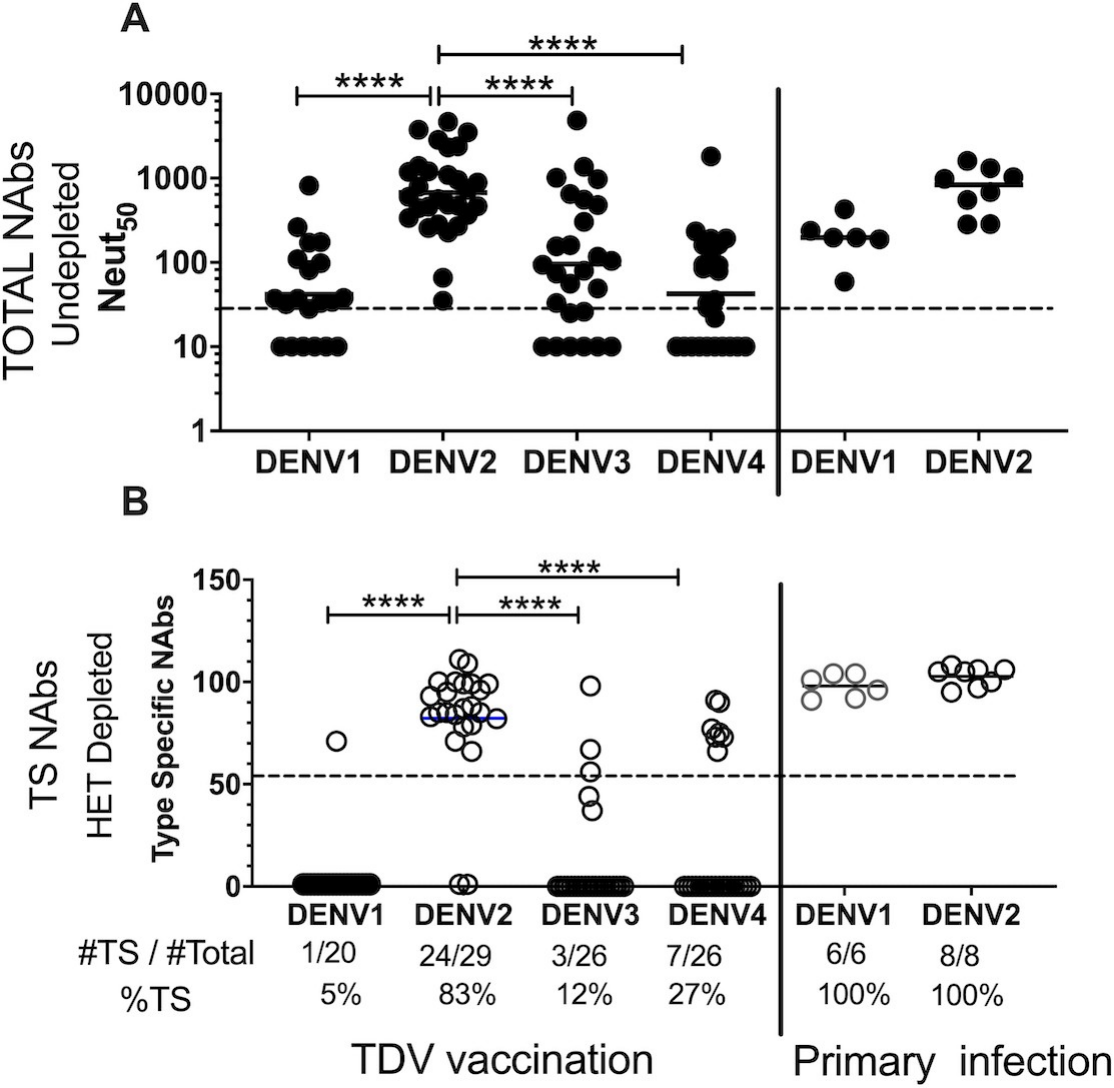
Analysis of the levels of TS nAbs in a subset of DEN-205 clinical samples that met enhanced inclusion criteria for TS analysis. Samples were included in this analysis only if the efficiency of Ab depletion for the serotype used as heterologous was >90%, or if the HET-depleted titer was <20. Only 20 subjects met the criterium for DV1 analysis, 29 for DV2, 26 for DV3 and 26 for DV4. The DENV1 and DENV2 primary infection samples are the same as in Fig 2. (A) Total NAb titers in undepleted sera. (B) Estimated levels of TS nAbs using the formula [(log10 Neut50 heterologous depletion/log10Neut50 BSA depletion)x100]. The dotted line in A is the lowest dilution tested. The dotted line in B represents the set cutoff value (55) for evidence of TS nAbs. The number of samples with TS nAbs for each serotype is indicated below each graph as a ratio over the total # of samples analyzed. Statistical analysis was done using nonparametric Mann-Whitney test between serotype 2 and the other serotypes in the TDV vaccination group. **** P < 0.0001.

We also performed a more stringent analysis to estimate levels of TS nAbs that took into consideration the efficiency of antibody depletion. In some individuals with high titers of antibodies after vaccination (mostly DENV2 Abs), it was difficult to remove all antigen-specific antibodies using the beads coated with purified virions. As incomplete removal of cross-reactive antibodies targeting epitopes conserved between serotypes can result in overestimating levels of TS nAbs, here we only included samples that showed >90% removal of cross reactive antibodies. We also included samples with control-depleted titers of < 40, which were considered negative for TS nAbs. The number of samples that met the acceptance criterium were 20/30 for DENV1, 29 for DENV2, 26 for DENV3 and 26 for DENV4. Fig 3 shows the Neut50 titers after BSA control depletions for those samples that met the acceptance criteria for TS analysis. We found DENV1 TS nAbs in 1/20 (5%), DENV2 TS nAbs in 24/29 (83%), DENV3 TS nAbs in 3/26 (12%) and DENV4 TS nAbs in 7/26 (27%). In contrast, all the control subjects exposed to WT primary DENV1 or DENV2 infection developed high levels of TS nAbs (Fig 3).

In summary, the results generated by both analyses (Figs 2 and 3) are consistent and show that in humans TAK-003 reliably elicited TS nAbs to DENV2 (77–83%), while TS nAbs to DENV1, DENV3 and DENV4 were detected at lower level in a subset of individuals (5–27%) who were vaccinated.

### Mapping the DV2 TS nAb response in NHP and in humans

Having established that TAK-003 reliably induced TS nAbs to DENV2 in people and monkeys, next we mapped epitopes on DENV2 recognized by those TS nAbs. Previous studies by our group have demonstrated that some TS nAbs elicited after DENV2 natural infection map to quaternary antigenic regions/epitopes involving EDIII of the E protein [30,34]. We used a recombinant chimeric rDENV4/2 virus displaying the DENV2 TS epitopes centered on EDIII on a DENV4 E protein backbone to map vaccine responses. Gain of neutralization by the chimeric DENV4/2 virus over the parental DENV4 backbone was evidence of DENV2 neutralizing Abs targeting sites in EDIII. The properties of the chimeric and parental viruses used in this experiment are described elsewhere [30,34].

**Fig 4:**
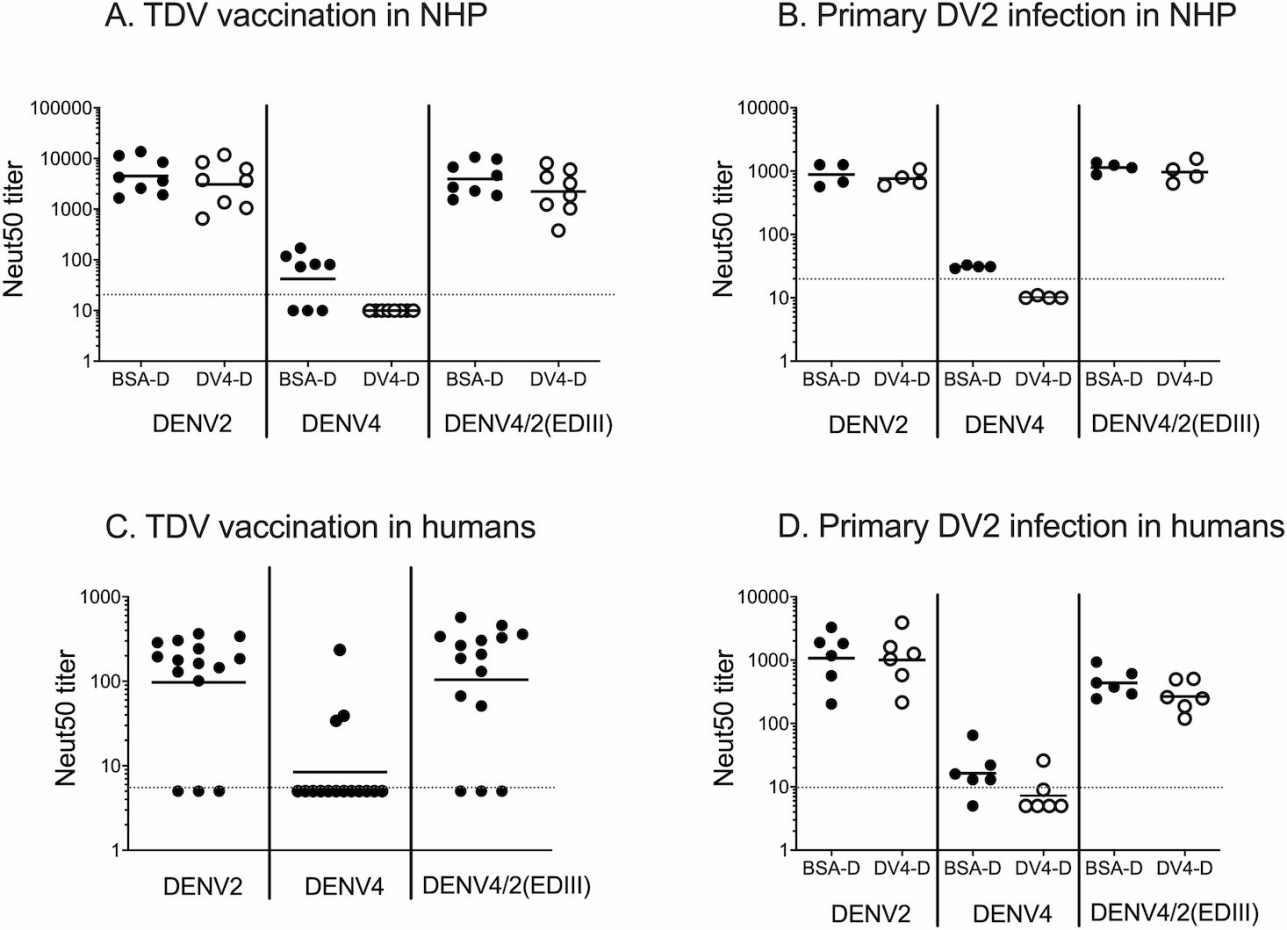
**Tracking the molecular specificity of DV2 TS nAbs to epitopes on EDIII in sera from TDV vaccinated NHPs (A), WT DV2 infected NHPs (B), TDV vaccinated, baseline seronegative adults (C) or adults who had a DV2 natural infection (D).** Neut50 titers to parental viruses DV2 (EDIII epitope donor), DV4 (backbone recipient of epitope transplant) and chimeric virus DV4/2EDIII (DV2 EDIII transplanted into DV4 E protein) were determined in sera collected 180 days after infection or vaccination (A, B, C). Sera used in (D) were collected in subjects pre-TDV vaccination, with evidence of past primary DENV2 infection based on Neut50 titers to DV2 > 4-fold higher than those to the other 3 serotypes. (A) (B) and (D) show titers in control-depleted (BSA-D) and DV4-depleted sera. (C) shows titers in undepleted sera. Horizontal lines and error bars represent GMT +/- 95% CI. Dotted line represents lower limit of detection.

Sera from NHP were tested for neutralization against DENV4/2, DENV2 and DENV4 viruses (Fig 4A). In order to assess gain of neutralization to the transplanted epitope in the chimeric virus, we had to deplete the sera of DENV4 binding Abs to just measure responses directed to the transplanted DENV2 domain. After removal of DENV4 binding Abs, the vaccinated monkeys maintained high titers to DENV2 (Neut50 GMT = 3077), did not neutralize DENV4 (Neut50 < 10) and efficiently neutralized the DENV4/2 virus chimera (Neut50 GMT = 2223) (Fig 4A). A similar pattern was observed in 4 monkeys infected with WT DENV2 (Fig 4B).

Sera from 15 vaccinated humans were tested for neutralization of DENV2, DENV4 and DENV4/2 viruses (Fig 4C). As most subjects had very low undatable levels of DENV4 nAbs, we did not remove DENV4 binding antibodies before performing the neutralization assays. Twelve of the 15 subjects tested neutralized DENV4/2 (GMT = 104) better than DENV4 alone, further implicating EDIII as a target of neutralizing Abs. We performed depletions and neutralizations as described for Fig 4B to map antibodies induced after a primary DENV2 infection in humans (Fig 4D). As reported earlier [30], our results show that EDIII is an important target of DENV2 type specific neutralizing Abs after primary DENV2 infection in humans. We concluded that most subjects vaccinated with TAK-003 develop DENV2 TS nAbs that map to epitopes centered on EDIII, which is a major target of nAbs induced by WT DENV2 infections [30,34].

## Discussion

The development of tetravalent dengue vaccines has been guided by nAbs to each serotype as a correlate of safe and effective vaccine induced immunity. However, the presence of nAbs alone to each serotype has proven to be an unreliable correlate of protection [19]. In baseline dengue seronegative people who receive live-attenuated tetravalent DENV vaccines, TS-nAb, which is a measure of immunity independently stimulated by each vaccine component, may be a better correlate of protection [24,35].

In a previous study, we analyzed specimens from Takeda studies 104 and 203 to understand the specificity of vaccine induced serum nAbs in individuals who were DENV seronegative or seropositive at baseline [36]. In individuals who were seronegative at baseline, we observed high levels of DENV2 nAbs that tracked with TS epitopes on serotype 2. Our results were in agreement with the DENV2 vaccine component replicating and independently stimulating immunity in study subjects. The results for DENV1, 3 and 4 were mixed and harder to interpret. Overall levels of DENV1, 2 and 4 nAbs were about 10-fold lower than the DENV2 titers. Moreover, only about half the study subjects had sufficient levels of DENV1, 3 or 4 nAbs for mapping the responses. Mapping studies with epitope transplant chimeric DENVs and Ab blockade assays indicated that a few individuals did develop nAbs that recognized unique epitopes on DENV1 or 3. However, these conclusions were subject to alternate explanations because we did not use Ab depletion strategies to isolate and characterize TS serum Abs. To overcome this ambiguity about the specificity of TAK-003 induced DENV1, 3 and 4 neutralizing antibodies, in the current study we used antibody depletion methods to remove DENV cross-reactive antibodies before determining the level and specificity of type-specific antibodies.

In the current analysis of 30 seronegative subjects who received TAK-003, most subjects (83%) had high levels of DENV2 TS-nAbs that tracked with known epitopes on DENV2. In contrast, the nAb levels to the other three serotypes were lower and 5%, 12% and 27% of subjects had TS nAbs to DENV1, 3 and 4 respectively. NHPs that received TAK-003 had high levels of DENV2 TS nAbs and low or no detectable TS nAbs to DENV1 or 3. Together, these results indicate that TAK-003 stimulates an independent and type-specific DENV2 nAb response in subjects who were seronegative at baseline. In contrast, most subjects developed lower levels of mainly DENV cross-reactive nAbs to the other three serotypes that are likely derived from the dominant DENV2 vaccine component.

Our conclusion that antibodies stimulated by TAK-003 are mainly derived from the dominant DENV2 is supported by other studies to characterize the vaccine in animals and humans [27,28, 29]. Soon after vaccination, investigators have readily detected replicating DENV2 vaccine virus or viral RNA and rarely detected the other three vaccine components [28,29]. More recently, Biswal et al. analyzed nAb levels in 702 DENV seronegative subjects who received two doses of the vaccine and observed that DENV2 Nab titers were 10 fold or more higher than the DENV1, 3, 4 titers [25].

Phase III clinical trials of TAK-003 are currently ongoing. Results of vaccine efficacy at 12 and 18 months after vaccination have been published [25,26]. In individuals seronegative at baseline, overall vaccine efficacy (VE) was 66% at 18 months after vaccination. In this population, VE differed greatly by serotype. VE was high against DENV2 (98%), moderate against DENV1 (66%) and the vaccine was not efficacious against DENV3. The number of DENV4 cases was too low to estimate VE. These results are consistent with our hypothesis that serotype-specific neutralizing antibodies are a better predictor of vaccine efficacy than total neutralizing antibodies. In the current study most (>80%) baseline seronegative individuals who were vaccinated developed DENV2 type-specific, quaternary epitope targetting neutralizing antibodies and vaccine efficacy was highest against this serotype at 18 months. Only 12% of the individuals had evidence for DENV3 type-specific neutralizing antibodies and there is no efficacy against this serotype in the TAK-003 trial. While only 5% of individuals developed DENV1 type-specific neutralizing antibodies, VE was 66% against this serotype. The data for DENV1 indicates a role for immune mechanisms other than type-specific antibodies in protective immunity. Primary wild type DENV infections result in a period of transient serotype cross protective immunity that can extend to 12–24 months [37]. For the TAK-003 clinical trial, 12–18 months is too early to distinguish vaccine induced transient protection from long-term protection. As we learn more about the serotype specific VE of TAK-003 at 24 month or more after vaccination, we will be in a postion to better evaluate immune correlates of durable protection in DENV-naïve populations that receive the vaccine.

## Supporting information

S1 Fig Proportion of the total nAbs to each serotype contributed by TS nAbs after TDV vaccination and DENV1 or DENV2 primary infection. (PDF)

S1 Table Takeda TAK-003 vaccine formulations used in DEN-205. (PDF)

S2 Table Source of nonhuman primate serum samples. (PDF)

S3 Table Source of human serum samples. (PDF)

S4 Table Neut50 titers induced after one dose of TDV or HD-TDV in dengue naïve adults. (PDF)

S5 Table Neut50 titers in baseline sera from DENV1 and DENV2 immune subjects. (PDF)

S6 Table Post-depletion Neut50 titers and depletion efficiency in DEN-205 seronegative subjects after TAK-003 vaccination. (PDF)

## References

[1] Bhatt S, Gething PW, Brady OJ, Messina JP, Farlow AW, Moyes CL, et al. The global distribution and burden of dengue. Nature. 2013;496(7446):504–7. 10.1038/nature12060 23563266 PMC3651993

[2] Radke EG, Gregory CJ, Kintziger KW, Sauber-Schatz EK, Hunsperger EA, Gallagher GR, et al. Dengue outbreak in Key West, Florida, USA, 2009. Emerg Infect Dis. 2012;18(1):135–7. 10.3201/eid1801.110130 22257471 PMC3310087

[3] Schaffner F, Mathis A. Dengue and dengue vectors in the WHO European region: past, present, and scenarios for the future. Lancet Infect Dis. 2014;14(12):1271–80. 10.1016/S1473-3099(14)70834-5 25172160

[4] Montoya M, Gresh L, Mercado JC, Williams KL, Vargas MJ, Gutierrez G, et al. Symptomatic versus inapparent outcome in repeat dengue virus infections is influenced by the time interval between infections and study year. PLoS Negl Trop Dis. 2013;7(8):e2357. 10.1371/journal.pntd.0002357 23951377 PMC3738476

[5] Sabin AB. Research on dengue during World War II. Am J Trop Med Hyg. 1952;1(1):30–50. 10.4269/ajtmh.1952.1.30 14903434

[6] Halstead SB. Etiologies of the experimental dengues of Siler and Simmons. Am J Trop Med Hyg. 1974;23(5):974–82. 10.4269/ajtmh.1974.23.974 4615598

[7] Wahala WM, Silva AM. The human antibody response to dengue virus infection. Viruses. 2011;3(12):2374–95. 10.3390/v3122374 22355444 PMC3280510

[8] Katzelnick LC, Montoya M, Gresh L, Balmaseda A, Harris E. Neutralizing antibody titers against dengue virus correlate with protection from symptomatic infection in a longitudinal cohort. Proc Natl Acad Sci U S A. 2016;113(3):728–33. 10.1073/pnas.1522136113 26729879 PMC4725482

[9] de Alwis R, Beltramello M, Messer WB, Sukupolvi-Petty S, Wahala WM, Kraus A, et al. In-depth analysis of the antibody response of individuals exposed to primary dengue virus infection. PLoS Negl Trop Dis. 2011;5(6):e1188. 10.1371/journal.pntd.0001188 21713020 PMC3119640

[10] de Alwis R, Williams KL, Schmid MA, Lai CY, Patel B, Smith SA, et al. Dengue viruses are enhanced by distinct populations of serotype cross-reactive antibodies in human immune sera. PLoS Pathog. 2014;10(10):e1004386. 10.1371/journal.ppat.1004386 25275316 PMC4183589

[11] Appanna R, Kg S, Xu MH, Toh YX, Velumani S, Carbajo D, et al. Plasmablasts During Acute Dengue Infection Represent a Small Subset of a Broader Virus-specific Memory B Cell Pool. EBioMedicine. 2016;12:178–88. 10.1016/j.ebiom.2016.09.003 27628668 PMC5078588

[12] Patel B, Longo P, Miley MJ, Montoya M, Harris E, de Silva AM. Dissecting the human serum antibody response to secondary dengue virus infections. PLoS Negl Trop Dis. 2017;11(5):e0005554. 10.1371/journal.pntd.0005554 28505154 PMC5444852

[13] Dejnirattisai W, Wongwiwat W, Supasa S, Zhang X, Dai X, Rouvinski A, et al. A new class of highly potent, broadly neutralizing antibodies isolated from viremic patients infected with dengue virus. Nat Immunol. 2015;16(2):170–7. 10.1038/ni.3058 25501631 PMC4445969

[14] Tsai WY, Lai CY, Wu YC, Lin HE, Edwards C, Jumnainsong A, et al. High-avidity and potently neutralizing cross-reactive human monoclonal antibodies derived from secondary dengue virus infection. J Virol. 2013;87(23):12562–75. 10.1128/JVI.00871-13 24027331 PMC3838129

[15] Weiskopf D, Angelo MA, Sidney J, Peters B, Shresta S, Sette A. Immunodominance changes as a function of the infecting dengue virus serotype and primary versus secondary infection. J Virol. 2014;88(19):11383–94. 10.1128/JVI.01108-14 25056881 PMC4178794

[16] de Silva AM, Harris E. Which Dengue Vaccine Approach Is the Most Promising, and Should We Be Concerned about Enhanced Disease after Vaccination? The Path to a Dengue Vaccine: Learning from Human Natural Dengue Infection Studies and Vaccine Trials. Cold Spring Harb Perspect Biol. 2018;10(6). 10.1101/cshperspect.a029371 PMC5983190 28716891

[17] Capeding MR, Tran NH, Hadinegoro SR, Ismail HI, Chotpitayasunondh T, Chua MN, et al. Clinical efficacy and safety of a novel tetravalent dengue vaccine in healthy children in Asia: a phase 3, randomised, observer-masked, placebo-controlled trial. Lancet. 2014;384(9951):1358–65. 10.1016/S0140-6736(14)61060-6 25018116

[18] Hadinegoro SR, Arredondo-Garcia JL, Capeding MR, Deseda C, Chotpitayasunondh T, Dietze R, et al. Efficacy and Long-Term Safety of a Dengue Vaccine in Regions of Endemic Disease. N Engl J Med. 2015;373(13):1195–206. 10.1056/NEJMoa1506223 26214039

[19] Sridhar S, Luedtke A, Langevin E, Zhu M, Bonaparte M, Machabert T, et al. Effect of Dengue Serostatus on Dengue Vaccine Safety and Efficacy. N Engl J Med. 2018;379(4):327–40. 10.1056/NEJMoa1800820 29897841

[20] Barban V, Mantel N, De Montfort A, Pagnon A, Pradezynski F, Lang J, et al. Improvement of the Dengue Virus (DENV) Nonhuman Primate Model via a Reverse Translational Approach Based on Dengue Vaccine Clinical Efficacy Data against DENV-2 and -4. J Virol. 2018;92(12). 10.1128/JVI.00440-18 PMC5974474 29593041

[21] Guirakhoo F, Pugachev K, Zhang Z, Myers G, Levenbook I, Draper K, et al. Safety and efficacy of chimeric yellow Fever-dengue virus tetravalent vaccine formulations in nonhuman primates. J Virol. 2004;78(9):4761–75. 10.1128/jvi.78.9.4761-4775.2004 15078958 PMC387722

[22] Guy B. Immunogenicity of sanofi pasteur tetravalent dengue vaccine. J Clin Virol. 2009;46 Suppl 2:S16–9. 10.1016/S1386-6532(09)70290-2 19800561

[23] Guy B, Barban V, Mantel N, Aguirre M, Gulia S, Pontvianne J, et al. Evaluation of interferences between dengue vaccine serotypes in a monkey model. Am J Trop Med Hyg. 2009;80(2):302–11. 19190230

[24] Henein S, Swanstrom J, Byers AM, Moser JM, Shaik SF, Bonaparte M, et al. Dissecting Antibodies Induced by a Chimeric Yellow Fever-Dengue, Live-Attenuated, Tetravalent Dengue Vaccine (CYD-TDV) in Naive and Dengue-Exposed Individuals. J Infect Dis. 2017;215(3):351–8. 10.1093/infdis/jiw576 27932620 PMC6392503

[25] Biswal S, Reynales H, Saez-Llorens X, Lopez P, Borja-Tabora C, Kosalaraksa P, et al. Efficacy of a Tetravalent Dengue Vaccine in Healthy Children and Adolescents. N Engl J Med. 2019;381(21):2009–19. 10.1056/NEJMoa1903869 31693803

[26] Biswal S, Borja-Tabora C, Martinez Vargas L, Velasquez H, Theresa Alera M, Sierra V, et al. Efficacy of a tetravalent dengue vaccine in healthy children aged 4–16 years: a randomised, placebo-controlled, phase 3 trial. Lancet. 2020. 10.1016/S0140-6736(20)30414-1 32197105

[27] Osorio JE, Brewoo JN, Silengo SJ, Arguello J, Moldovan IR, Tary-Lehmann M, et al. Efficacy of a tetravalent chimeric dengue vaccine (DENVax) in Cynomolgus macaques. Am J Trop Med Hyg. 2011;84(6):978–87. 10.4269/ajtmh.2011.10-0592 21633037 PMC3110349

[28] Rupp R, Luckasen GJ, Kirstein JL, Osorio JE, Santangelo JD, Raanan M, et al. Safety and immunogenicity of different doses and schedules of a live attenuated tetravalent dengue vaccine (TDV) in healthy adults: A Phase 1b randomized study. Vaccine. 2015;33(46):6351–9. 10.1016/j.vaccine.2015.09.008 26384447

[29] Tricou V, Low JG, Oh HM, Leo YS, Kalimuddin S, Wijaya L, et al. Safety and immunogenicity of a single dose of a tetravalent dengue vaccine with two different serotype-2 potencies in adults in Singapore: A phase 2, double-blind, randomised, controlled trial. Vaccine. 2020;38(6):1513–9. 10.1016/j.vaccine.2019.11.061 31843269

[30] Gallichotte EN, Widman DG, Yount BL, Wahala WM, Durbin A, Whitehead S, et al. A new quaternary structure epitope on dengue virus serotype 2 is the target of durable type-specific neutralizing antibodies. mBio. 2015;6(5):e01461–15. 10.1128/mBio.01461-15 26463165 PMC4620467

[31] Swanstrom JA, Nivarthi UK, Patel B, Delacruz MJ, Yount B, Widman DG, et al. Beyond Neutralizing Antibody Levels: The Epitope Specificity of Antibodies Induced by National Institutes of Health Monovalent Dengue Virus Vaccines. J Infect Dis. 2019;220(2):219–27. 10.1093/infdis/jiz109 30895307 PMC6581895

[32] Collins MH, McGowan E, Jadi R, Young E, Lopez CA, Baric RS, et al. Lack of Durable Cross-Neutralizing Antibodies Against Zika Virus from Dengue Virus Infection. Emerg Infect Dis. 2017;23(5):773–81. 10.3201/eid2305.161630 28418292 PMC5403059

[33] Sariol CA, White LJ. Utility, limitations, and future of non-human primates for dengue research and vaccine development. Front Immunol. 2014;5:452. 10.3389/fimmu.2014.00452 25309540 PMC4174039

[34] Gallichotte EN, Baric TJ, Yount BL Jr, Widman DG, Durbin A, Whitehead S, et al. Human dengue virus serotype 2 neutralizing antibodies target two distinct quaternary epitopes. PLoS Pathog. 2018;14(2):e1006934. 10.1371/journal.ppat.1006934 29481552 PMC5843351

[35] Nivarthy U; Durbin A; Whitehead S; Kirkpatrick B; Pierce K; Diehl S; Katzelnick L; Baric R; deSilva A. A tetravalent live attenuated dengue virus vaccine stimulates balanced immunity to multiple serotypes in humans. Accepted in Nature Communications. 2021. 10.1038/s41467-021-21384-0 PMC7889627 33597521

[36] Swanstrom JA, Henein S, Plante JA, Yount BL, Widman DG, Gallichotte EN, et al. Analyzing the Human Serum Antibody Responses to a Live Attenuated Tetravalent Dengue Vaccine Candidate. J Infect Dis. 2018;217(12):1932–41. 10.1093/infdis/jiy063 29800370 PMC5972589

[37] Anderson KB, Gibbons RV, Cummings DA, Nisalak A, Green S, Libraty DH, et al. A shorter time interval between first and second dengue infections is associated with protection from clinical illness in a school-based cohort in Thailand. J Infect Dis. 2014;209(3):360–8. 10.1093/infdis/jit436 23964110 PMC3883164

