# Supplementary material for: Defining levels of dengue virus serotype-specific neutralizing antibodies induced by a live attenuated tetravalent dengue vaccine (TAK-003)
Source: PLoS Negl Trop Dis. 2021 Mar 12;15(3):e0009258. doi: 10.1371/journal.pntd.0009258 (PMC7990299; doi:10.1371/journal.pntd.0009258)
Supplement: S1 Fig — (PDF) [file pntd.0009258.s001.pdf]

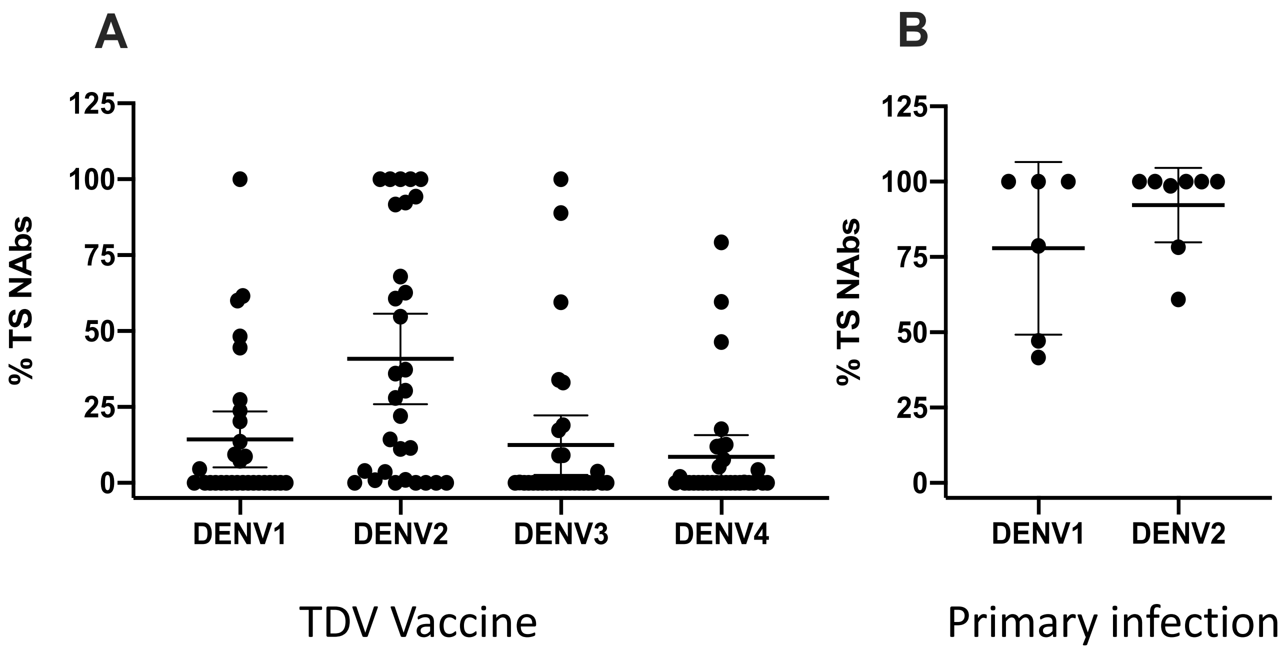

S1 Fig. Proportion of the total NAbs to each serotype contributed by TS NAbs after (A) TDV vaccination and (B) DENV1 or DENV2 primary infection in humans. The levels of TS nAbs measured in figure 2B are expressed here as the % of the total nAbs shown in figure 2A. (A) Vaccinated subjects; (B) Subjects with primary infection. The horizontal bar represents the mean percentage of TS nAbs with 95% CI.
