# Supplementary material for: Defining levels of dengue virus serotype-specific neutralizing antibodies induced by a live attenuated tetravalent dengue vaccine (TAK-003)
Source: PLoS Negl Trop Dis. 2021 Mar 12;15(3):e0009258. doi: 10.1371/journal.pntd.0009258 (PMC7990299; doi:10.1371/journal.pntd.0009258)
Supplement: S1 Table — (PDF) [file pntd.0009258.s002.pdf]

S1 Table. Takeda Vaccine formulations used in DEN-205

Formulation	FFU/dose			
	TDV1	TDV2	TDV3	TDV4
TDV*	2×10^4	5×10^3	1×10^5	3×10^5
HD-TDV	2×10^4	5×10^4	1×10^5	3×10^5

* Used in ongoing Phase 3 trials
