# Supplementary material for: Defining levels of dengue virus serotype-specific neutralizing antibodies induced by a live attenuated tetravalent dengue vaccine (TAK-003)
Source: PLoS Negl Trop Dis. 2021 Mar 12;15(3):e0009258. doi: 10.1371/journal.pntd.0009258 (PMC7990299; doi:10.1371/journal.pntd.0009258)
Supplement: S2 Table — (PDF) [file pntd.0009258.s003.pdf]

S2 Table. Source of nonhuman primate serum samples

Study	Serotype Analyzed	Treatment Group	N	Vaccination or infection day	Day of serum collection
Takeda study DNHP007 Tetravalent TDV vaccine	DV1	One dose TDV	4	0	180
		Two doses TDV	4	0, 180	180 post dose 2
		WT DV1 infection	6	0	180
	DV2	One dose TDV	4	0	180
		Two doses TDV	4	0, 180	180 post dose 2
		WT DV2 infection	6	0	180
	DV3	One dose TDV	4	0	180
		Two doses TDV	4	0, 180	180 post dose 2
		WT DV3 infection	6	0	180
