# Supplementary material for: Defining levels of dengue virus serotype-specific neutralizing antibodies induced by a live attenuated tetravalent dengue vaccine (TAK-003)
Source: PLoS Negl Trop Dis. 2021 Mar 12;15(3):e0009258. doi: 10.1371/journal.pntd.0009258 (PMC7990299; doi:10.1371/journal.pntd.0009258)
Supplement: S3 Table — (PDF) [file pntd.0009258.s004.pdf]

S3 Table. Source of human serum samples

Study	Serostatus at vaccination	Vaccine (One dose)	N	Day of serum collection
Takeda study DEN-205 Phase 2 trial Singapore Adults 21-45 Years Old (N=400)	Dengue seronegative	TV TDV	14	180 days post vaccination
		TV HD-TDV	16	180 days post vaccination
	Dengue 1 seropositive	-	6	Day 0 pre-vaccination
	Dengue 2 seropositive	-	8	Day 0 pre-vaccination
