# Supplementary material for: Defining levels of dengue virus serotype-specific neutralizing antibodies induced by a live attenuated tetravalent dengue vaccine (TAK-003)
Source: PLoS Negl Trop Dis. 2021 Mar 12;15(3):e0009258. doi: 10.1371/journal.pntd.0009258 (PMC7990299; doi:10.1371/journal.pntd.0009258)
Supplement: S4 Table — (PDF) [file pntd.0009258.s005.pdf]

S4 Table. $Neut_{50}$ titers in baseline sera from DENV1 and DENV2 immune subjects

	$NEUT_{50}$ TITERS			
	**DENV1**	**DENV2**	**DENV3**	**DENV4**
**DENV1 immune sera**	**216**	79	10	10
	**271**	85	45	41
	**140**	50	38	20
	**465**	20	20	20
	**384**	43	76	20
	**612**	59	168	20
**DENV2 immune sera**	37	**1512**	20	29
	45	**1317**	40	42
	39	**982**	20	41
	74	**1024**	105	89
	20	**553**	20	51
	56	**686**	20	53
	22	**283**	20	44
	133	**285**	28	74
