# Supplementary material for: Defining levels of dengue virus serotype-specific neutralizing antibodies induced by a live attenuated tetravalent dengue vaccine (TAK-003)
Source: PLoS Negl Trop Dis. 2021 Mar 12;15(3):e0009258. doi: 10.1371/journal.pntd.0009258 (PMC7990299; doi:10.1371/journal.pntd.0009258)
Supplement: S5 Table — (PDF) [file pntd.0009258.s006.pdf]

S5 Table. Comparison of Neut50 titers induced after one dose of TDV or HD-TDV in dengue seronegative adults

	DV1		DV2		DV3		DV4	
	**TDV**	**HD-TDV**	**TDV**	**HD-TDV**	**TDV**	**HD-TDV**	**TDV**	**HD-TDV**
	69	181	366	2844	171	94	161	125
	98	263	283	459	80	481	79	162
	421	205	789	891	955	1360	193	88
	174	172	271	1187	555	49	234	93
	94	802	2319	4642	4857	160	163	192
	81	224	467	3503	117	650	605	329
	819	109	2386	1203	970	304	1817	93
	38	157	66	1395	74	155	10	259
	28	50	338	729	104	1013	10	36
	10	10	35	952	10	10	10	10
	32	10	474	227	72	26	85	10
	10	34	483	1090	25	10	29	22
	95	33	41	601	33	80	10	33
	10	37	564	3754	10	56	10	10
		10		257		10		10
		37		440		10		10
GMT	**61**	**71**	**336**	**1032**	**123**	**90**	**65**	**47**
