# Supplementary material for: Defining levels of dengue virus serotype-specific neutralizing antibodies induced by a live attenuated tetravalent dengue vaccine (TAK-003)
Source: PLoS Negl Trop Dis. 2021 Mar 12;15(3):e0009258. doi: 10.1371/journal.pntd.0009258 (PMC7990299; doi:10.1371/journal.pntd.0009258)
Supplement: S6 Table — (PDF) [file pntd.0009258.s007.pdf]

S6 Table. Post-depletion Neut50 titers and depletion efficiencies in DEN-205 seronegative subjects after TAK-003 vaccination

DENV-1				DENV-2				DENV-3				DENV-4			
Neut50			% DV1 Abs Depleted	Neut50			% DV2 Abs Depleted	Neut50			% DV3 Abs Depleted	Neut50			% DV4 Abs Depleted
CONTROL Depleted	HOMOL. SEROTYPE Depleted	HETEROL. SEROTYPE Depleted		CONTROL Depleted	HOMOL. SEROTYPE Depleted	HETEROL. SEROTYPE Depleted		CONTROL Depleted	HOMOL. SEROTYPE Depleted	HETEROL. SEROTYPE Depleted		CONTROL Depleted	HOMOL. SEROTYPE Depleted	HETEROL. SEROTYPE Depleted	
	(DV1+DV3)*	(DV2+DV4)*			(DV2+DV4)	(DV1+DV3)			(DV1+DV3)	(DV2+DV4)			(DV2+DV4)	(DV1+DV3)	
181	10	26	94	2844	333	423	88	94	10	10	89	125	10	10	92
69	10	22	86	366	57	466	84	171	25	53	85	161	80	95	50
98	10	10	90	283	10	10	96	80	10	10	88	79	10	10	87
421	10	29	98	789	21	10	97	955	96	381	90	193	39	45	80
174	10	10	94	271	10	20	96	555	10	10	98	234	20	37	91
94	10	33	89	2319	1161	1887	50	4857	10	10	100	163	50	50	69
263	10	10	96	459	29	184	94	481	10	10	98	162	10	10	94
205	10	27	95	891	107	195	88	1360	24	25	98	88	24	10	73
172	10	10	94	1187	528	353	56	49	10	10	80	93	10	10	89
802	10	68	99	4642	221	261	95	160	10	10	94	192	27	47	86
81	10	10	88	467	28	294	94	117	10	10	91	605	417	441	31
224	31	25	86	3503	160	1177	95	650	10	10	98	329	62	77	81
109	10	10	91	1203	116	522	90	304	10	10	97	93	28	30	70
157	10	45	94	1395	262	1441	81	155	10	10	94	259	79	37	69
819	10	120	99	2386	234	256	90	970	10	45	99	1817	176	937	90
38	10	27	73	66	10	10	85	74	10	10	86	10	10	10	0
50	10	34	80	729	82	225	89	1013	10	102	99	36	22	33	40
28	10	10	65	338	35	315	90	104	22	95	79	10	10	10	0
10	10	10	0	35	21	10	38	10	10	10	0	10	10	10	0
10	10	10	0	952	131	225	86	10	10	10	0	10	10	27	0
10	10	10	0	227	106	407	53	26	10	42	61	10	10	10	0
34	10	69	70	1090	137	1036	87	10	10	10	0	22	10	10	54
32	10	10	69	474	156	819	67	72	10	31	86	85	10	55	88
10	10	10	0	483	58	177	88	25	10	10	59	29	10	10	66
33	10	10	70	601	187	567	69	80	10	22	88	33	10	10	70
95	10	51	89	41	25	23	38	33	10	24	70	10	10	10	0
37	24	30	35	3754	630	1079	83	56	41	10	27	10	10	10	0
10	22	10	0	564	266	429	53	10	10	10	0	10	10	10	0
10	10	10	0	257	21	300	92	10	10	10	0	10	10	10	0
37	10	10	73	440	76	323	83	10	10	10	0	10	10	10	0

* DENV used in depletion
